# Anomalous thermoelectricity in strained Bi_2_Te_3_ films

**DOI:** 10.1038/srep32661

**Published:** 2016-09-07

**Authors:** Yucong Liu, Jiadong Chen, Huiyong Deng, Gujin Hu, Daming Zhu, Ning Dai

**Affiliations:** 1 National Laboratory for Infrared Physics, Shanghai Institute of Technical Physics, Chinese Academy of Sciences, Shanghai 200083, China; 2 University of Chinese Academy of Science, Beijing 100049, China; 3 Changzhou Institute of Optoelectronic Technology, Changzhou 213164, China; 4 Department of Physics, University of Missouri-Kansas City, Missouri 64110, USA; 5 Jiangsu Collaborative Innovation Center of Photovolatic Science and Engineering, Changzhou 213164, China

## Abstract

Bi_2_Te_3_-based alloys have been intensively used for thermoelectric coolers and generators due to their high Seebeck coefficient *S.* So far, efforts to improve the *S* have been made mostly on changing the structures and components. Herein, we demonstrate an anomalous thermoelectricity in strained Bi_2_Te_3_ films, *i.e.*, the value of *S* is obviously changed after reversing the direction of temperature gradient. Further theoretical and experimental analysis shows that it originates from the coupling of thermoelectric and flexoelectric effects caused by a stress gradient. Our finding provides a new avenue to adjust the *S* of Bi_2_Te_3_-based thermoelectric materials through flexoelectric polarization.

As a typical 3D topological insulator, Bismuth Telluride (Bi_2_Te_3_) and its alloys have recently attracted significant interests due to their unique layered structures^1^,^2^,^3^. Actually, they are also famous thermoelectric materials with high thermoelectric coefficient near room temperature (RT) for applications in waste-heat recovery, refrigeration or portable power generation, since they possess notable properties such as a prominent Seebeck effect, low thermal conductivity and high value of the carrier concentration in RT^4^,^5^,^6^,^7^,^8^. The thermoelectric performance of materials is evaluated by figure of merit *ZT* = (*S*^2^*σ*/*k*)*T*, where *S* is the Seebeck coefficient, *σ* is the electrical conductivity, *k* is the thermal conductivity, and *T* is the absolute temperature^8^,^9^,^10^,^11^,^12^,^13^,^14^. In the past decades, many kinds of methods have been developed to improve the *ZT* value through modulating the parameters *S*, *σ* and *k* in order to improve the conversion efficiency of thermoelectric devices^10^,^13^,^15^. For example, donor or acceptor impurities and other elements are doping into the alloys in order to optimize the charge carrier concentration, but they usually increase the *k* at the same time^5^,^16^,^17^. And the solution derived nanostructured bulk materials are expected to reduce the lattice thermal conductivity by phonon scattering on the boundaries of nano-sized grains^18^, however they still show a low *ZT* value because of the poor *σ*^8^,^11^,^19^. So the challenge of improving *ZT* value is that these three parameters are mutually constrained, *e.g.* both *S* and *σ* are influenced by Fermi level and changing oppositely, and increasing *σ* leads to the increasing of *k* at the same time because of the Wiedemann-Franz law^19^. In recently years, Bi_2_Te_3_ based superlattices and quantum wires have been demonstrated to have a great potential in improving *ZT* value due to the enhancement of thermoelectric power and reduction of lattice thermal conductivity by phonon boundary scattering^7^,^20^,^21^. For example, Venkatasubramanian *et al*. have recently reported extremely high *ZT* value of 2.4 in *p*-type Bi_2_Te_3_/Sb_2_Te_3_ superlattices and 1.4 in n-type Bi_2_Te_3_/Bi_2_Te_2.83_Se_0.17_ superlattices, and the enhancement has been achieved by controlling the transport of phonons and electrons in superlattices^15^,^22^. In this work, anomalous thermoelectric effect is demonstrated in strained Bi_2_Te_3_ films grown on polyimide (PI), GaAs and InAs substrates, *i.e.* the *S* has been found to be dependent on the direction of temperature gradient and obvious difference has been observed when changing the direction. Moreover, we attributed this phenomenon to the coupling of thermoelectric and flexoelectric effects. And the results provide a different avenue to adjust the *S* with stress.

Bi_2_Te_3_ films on polyimide (PI) were fabricated by a modified hot wall epitaxy (HWE) method. Figure 1 gives the XRD pattern of the as-deposited Bi_2_Te_3_ films before and after annealing, in which four strong diffraction peaks occur. The most strongest peak comes from the (0, 0, 15) diffraction, and the other three peaks corresponds to (0, 0, 6), (0, 0, 18) and (0, 0, 21) diffractions, respectively. Hence, the XRD pattern is dominant by the diffraction peaks from the (0, 0, *l*) planes (*l* = 6, 15, 18, 21), indicating that the Bi_2_Te_3_ films are highly *c*-axis oriented. Additionally, two very weak peaks corresponding to (0, 1, 5) and (1, 0, 10) diffractions, respectively, are also observed, indicating that some disoriented crystallites exist in the film. The magnified (0, 0, 15) diffraction peaks are showed in Fig. S1, in which the shoulder peaks come from the diffraction of Cu Kα2 line. It is noted that the (0, 0, 15) diffraction intensity decreases after annealing, which implies that the crystalline quality of Bi_2_Te_3_ films by HWE method is sensitive to the growth conditions. The possible reason is that more crystal defects such as dislocations and grain boundaries are generated since PI substrates are gradually bended in annealing time. The full-width at half-maximum (FWHM) is about 0.1° and smaller than Ferhat *et al*.’s result (0.16°)^23^, indicating that our films have better crystalline quality. From the inset SEM images, it can be seen that the surface consists of closely coalesced crystal grains and many steps which are labeled by the colored zigzag lines to make them easily observed. These results indicating that these crystal grains have different thickness although each of them has a very smooth surface. Based on the XRD result, it is considered that these grains are single crystalline and *c*-axis oriented. Their average size is about 8 μm and larger than the reported size (about 4 μm), which implies that our experimental conditions improve the crystallinity of crystal grains, which is in consistent with the XRD result. It is also noted from the low-magnification SEM image that many disoriented crystal grains, one of which is labeled by the red rectangle, are observed, which are responsible for the weak (0, 1, 5) and (1, 0, 10) diffraction peaks in XRD results.

Prior to thermoelectric measurement, two golden electrodes were fabricated in the ends of the films symmetrically by argon ion sputtering, and then the film was placed on the center of a long rectangular graphite bar which has a good thermal conductivity. The temperature gradient was created by cooling one end of the bar with ice and heating the other end with a resistance heater, the temperature difference between two ends of the film was measured by two same thermocouples. In addition, the thermoelectric voltage (*V*_T_) was measured by Keithley 2182 Nano voltmeter. Figure 2 gives the dependence of thermoelectric voltage on temperature gradient (Δ*T*), measured at about 20 °C, in which the separated dots represent experimental data and both two lines are the linear fit results using least square method. It can be seen from curve (a) that the *V*_T_ almost increases linearly with Δ*T* and the fitted line agrees with experimental data very well, which all exhibit the typical characteristic of Seebeck effect. The Seebeck coefficient *S* is determined to be 204.38 *μV*/*K* from the slope of the fitted line and given in the inset. The positive value of *S* indicates that the Bi_2_Te_3_ film is *p*-type, ascribed to the existence of Bi_Te_ anti-site defect^24^,^25^, which is consistency with Hall measurement conclusion and the measured hole concentration is about 5 × 10^17^ cm^−3^. However, it is surprisingly found that one significant different Seebeck coefficient *S*_R_ is obtained after changing the direction of Δ*T*, which is showed by curve (b) labeled by symbol R. In order to eliminate the influence of experimental errors, *e.g.* a subtle difference of thermal conductivity between different directions of the substrate or sample stage, after the whole system took enough time to re-stabilize the temperature field and then the film was rotated 180 degrees horizontally *in situ*, we carried out the measurement again with all other conditions unchanged and found that both two measured *S* and *S*_R_ could be repeated very well. Therefore, our result conflicts with traditional Seebeck effect, for which one new *S*_R_ has not been obtained up to now even though changing the direction of Δ*T*. This implies that there possibly exists another internal electric field with a certain direction beside the thermoelectric field^26^.

The internal electric field usually exists in materials with the internal polarization, which could be induced by ferroelectricity, piezoelectricity and flexoelectricity. As we know that the crystal structure of Bi_2_Te_3_ (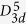 (R
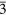
m) space group) does not belong to the known 20 piezoelectric crystal classes and there is no external electric field applied in the measurements, the internal polarization in Bi_2_Te_3_ films may not be induced by ferroelectricity or piezoelectricity. However, the flexoelectricity may be responsible for the internal polarization due to the strain gradient between films and PI substrates^27^,^28^. Actually, we have recently observed a phenomenon of stress-induced polarization in these strained Bi_2_Te_3_ films with *c*-axis oriented direction, showed in Fig. S2, which possibly originates from flexoelectricity. This mechanism is different with the piezoelectric effect recently found in monolayer MoS_2_ owing to the breaking of the inversion symmetry^29^,^30^,^31^. The flexoelectric effect can be introduced by the constitutive equation for electric polarization *P*_*i*_ due to mechanical strain.


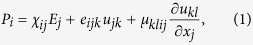


where *E*_*j*_, *u*_*jk*_ and 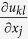 are the macroscopic electric field, strain tensor and its spatial gradient, respectively. Besides, *χ*_*ij*_ represents the dielectric tensor and *e*_*ijk*_ represents the piezoelectric tensor. The first two terms describes the dielectric and piezoelectric response and third term describes the flexoelectric response to a strain gradient. As flexoelectricity is described by a fourth-rank tensor *μ*_*klij*_, it is not limited to non-centrosymmetric structures, and its responds can be several orders of magnitude weaker than the piezoelectricity^27^. Hence, we attribute the above anomalous thermoelectricity to the coupling of thermoelectric and flexoelectric effects induced by the strain gradient between the films and substrates due to the thermal contraction of PI during the cooling process^32^. Figure 3 gives the measuring result of thermoelectric effect of Bi_2_Te_3_ films on InAs and GaAs substrates. It can be seen that the anomalous thermoelectricity is also observed, caused by the strain gradient between the film and substrate due to lattice mismatch, which further confirms our assumption^33^,^34^.

To clarify the involved physical mechanism in Figs 2 and 3, Fig. 4 schematically displays the movement of carries under both two effects, and the process of the electric field forming. The distribution of defects, carries and electric dipoles in the strained Bi_2_Te_3_ film is illustrated in Fig. 4(a). The electric dipoles come from the flexoelectric polarization, and the Bi_Te_ anti-site defect is a kind of intrinsic defect which is negatively charged and generate a hole in ionized state. Besides, there are also free electron hole pairs at room temperature due to its narrow band gap. Since the carriers’ concentration is relatively high in Bi_2_Te_3_ film, almost all electric dipoles are neutralized by the free carriers in stationary state, so the flexoelectric effect is too weak to be observed in this situation. When the temperature gradient is introduced into the system, however, the equilibrium state is broken, as illustrated in Fig. 4(b). As described by Seebeck effect, the hot end has a higher density of free carriers than the cold end, so they diffuse along the temperature gradient and are collected by electrode, which produces a positive voltage since the dominant carriers are holes. At the same time, the film becomes polarized as free carriers gradually escape from the electric dipoles, and the flexoelectric field (*E*_f_) is mainly determined by the carrier’s concentration just like the thermoelectric field (*E*_T_). Given the opposite direction, it can be expressed as


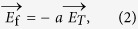


and *a* is a coefficient less than 1. In the balance state, the diffusion current density should equal with the drift current density, which is


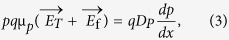


where *p* is the hole density, *q* is the electron charge, μ_*P*_ is the hole mobility, and *D*_*p*_ is the hole diffusion coefficient. According to Einstein relation *D*_*p*_/μ_*p*_ = *k*_0_*T*/*q*, the equation can be expressed as


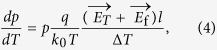


where *l* is the sample length, and Δ*T* is the temperature difference between sample ends. The hole density can be expressed as


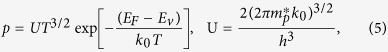


Substitute *p* in Eq. (4) gives


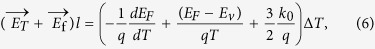


The thermoelectric voltage equals to the difference of Fermi levels over the charge *q*. However, it is noteworthy that the inclination of Fermi level is not equal to the inclination of band, since the temperature also takes effect. Furthermore, the inclination of band equals *q* multiplied by *E*_*T*_*l*. So the thermoelectric voltage can be expressed as


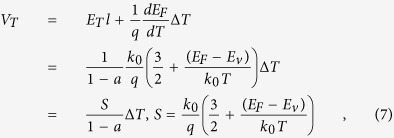


where *S* is the Seebeck coefficient. Equation (6) was used in the derivation of Eq. (7). Hence, according to Eq. (7), the *V*_T_ is linearly dependent on Δ*T*, and meanwhile due to the presence of the factor 1/(1−*a*), the measured *S* is larger than the normal, which is corresponding with the results of Fig. 2(a). After reversing the direction of temperature gradient, the effect of flexoelectricity is changed and the difference is illustrated in Fig. 4(c). In this case, the *E*_f_ has the same direction with *E*_T_, which turns the factor to 1/(1 + *a*) and causes the measured *S* smaller than the normal, corresponding with the results of Fig. 2(b).

To further confirm the existence of flexoelectric effect in strained Bi_2_Te_3_ films, the current-voltage (*I-V*) curves for Bi_2_Te_3_ film on 10 × 10 mm square PI substrate under different bending conditions were measured and compared sequentially. Since the Bi_2_Te_3_ is a very efficient thermoelectric material and a small temperature inhomogeneity will generate a voltage, the measurement was performed carefully to exclude the thermoelectric effect. First, the sample was placed on graphite which is very high thermal conductivity material in order to achieve the even temperature field; second, the sample was compressed by two glass slices which is poor thermal conductive and the whole system was settled in a dark and adiabatic environment; third, the test was carried out after the sample was bent and stabilized for enough time. The bending degree was controlled and characterized by the distance (*D*) between two glass slices, which was precisely measured by a caliper, and the results are illustrated in Fig. 5. All *I-V* curves are linear which indicates that Ohmic contacts between electrodes and films are formed and the Schottky barriers are absent. Figure 5(a) gives the *I-V* curves for flexible Bi_2_Te_3_ film in nature state and N-shape bend states. Since the PI substrate has a much larger coefficient of thermal expansion than the Bi_2_Te_3_ film, the sample shows arc slightly due to thermal contraction in the cooling process, and make the natural distance *D* is about 9 mm which is 10 mm originally^32^. As a result, the deposited film in nature is uneven compressively strained, and leads to the flexoelectric effect which is responsible for the derivation of the *I-V* curve of nature state from zero point of coordinate. After bending upward the sample to N_1_ (*D* = 6 mm), the strained film is released and even under an uneven tensile stress which makes the *I-V* curve shift to the opposite quadrant of the zero point. With further bending upward the sample to N_2_ (*D* = 3 mm), the film is under a larger tensile stress gradient and makes the *I-V* curve intercept a longer distance in *V*-axis than N_1_. Besides the translation of *I-V* curves, bending the film also decreases the slope value of *I-V* curves which means the increasing of resistance of Bi_2_Te_3_ films. Then the film is released and bending downward to U-shape, the related *I-V* curves are illustrated in Fig. 5(b). The *I-V* curve for U_1_ bending (*D* = 6 mm) shows a larger intercept and a smaller slope compared with the *I-V* curve in nature due to the increasing of compressive stress gradient in the film. And such changes become more apparent in *I-V* curve for U_2_ bending (*D* = 3 mm) owing to the further increasing of compressive stress gradient. One has to be mentioned here is that in each diagram of Fig. 5, the three *I-V* curves cross almost the same points, which may imply the resistances and open-circle voltages both increase with the external force increases in the same steps.

In summary, the values of Seebeck coefficient *S* of Bi_2_Te_3_ films on substrates of PI, GaAs, and InAs have been measured from two opposite directions at nearly room temperature, and are found to be directional dependent, indicating of an anomalous thermoelectricity. The flexoelectric effect caused by the strain gradient between films and substrates is proposed to be responsible for this phenomenon, and the existence of flexoelectricity in strained Bi_2_Te_3_ films is confirmed by *I-V* tests for films under different external forces. The involved mechanism of anomalous thermoelectricity is schematically illustrated, and the effect of flexoelectric field on thermal voltage has been discussed. The results indicate a new strategy of improving and modulation the *S* of the layered thermoelectric materials like Bi_2_Te_3_, and it also demonstrates the existence of prominent flexoelectric effect of Bi_2_Te_3_ from a new aspect.

## Methods

Bi_2_Te_3_ films were fabricated in a hot wall epitaxy (HWE) system, in which a resistance furnace was placed in a vacuum chamber and the substrate was heated by a halogen lamp. High pure Bi_2_Te_3_ powders (99.999%) were used as the evaporation source and the flexible polyimide (PI) was used as the substrate. The source temperature *T*_s_ and substrate temperature *T*_sub_ were set to 475 °C and 300 °C, respectively, and the growth time varies from 2 hours to 15 mins. The plasticizing temperature of PI is 400 °C which is higher than *T*_sub_. To improve the stability of resistivity, the films were annealed at 250 °C for 1 h after growth in the vacuum chamber^17^. The microstructures of the as-deposited Bi_2_Te_3_ films were characterized by the X-ray diffraction (XRD) and scanning electron microscopy (SEM).

The golden electrodes were fabricated by argon ion sputtering. The thermoelectric voltage (*V*_T_) was recorded by a Keithley 2182 nanovoltmeter, and the temperature gradient was measured by two Eurotherm 3504. The *I-V* curves were measured by a Keithley 2601A sourcemeter and Keithley 2182 nanovoltmeter. Hall measurements shows that the Bi_2_Te_3_ film is *p*-type conductive caused by Bi_Te_ anti-site defect^25^ and the measured hole concentration is about 5 × 10^17^ cm^−3^.

## Additional Information

**How to cite this article**: Liu, Y. *et al*. Anomalous thermoelectricity in strained Bi_2_Te_3_ films. *Sci. Rep.* **6**, 32661; doi: 10.1038/srep32661 (2016).

## Supplementary Material

Supplementary Information

## Figures and Tables

**Figure 1:**
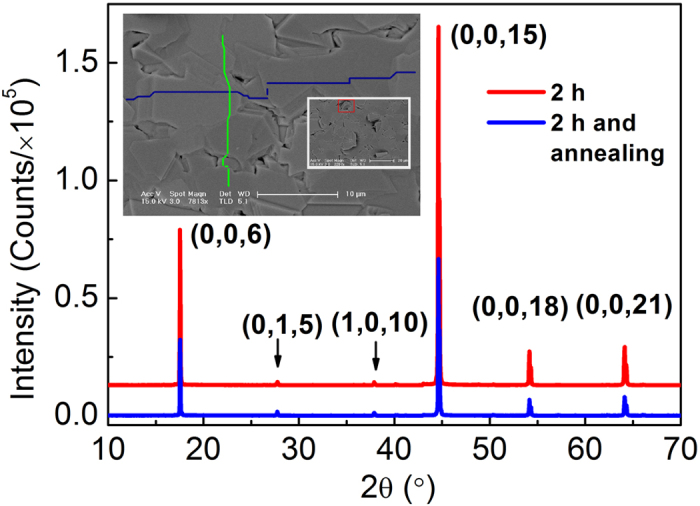
Microstructures of Bi_2_Te_3_ films on PI substrates by HWE method. The figure shows XRD pattern of the as-grown Bi_2_Te_3_ films before and after annealing. The inset shows SEM images of the film surface. Colored zigzag lines denote the steps and in the inset the purple rectangles represent disoriented crystal grains.

**Figure 2:**
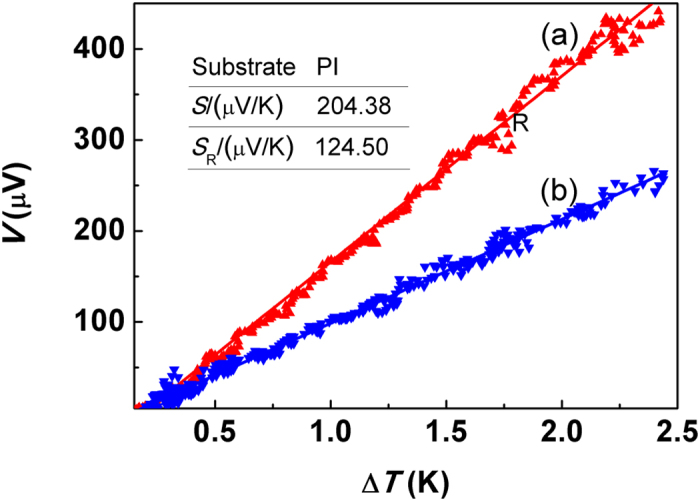
Thermoelectricity of strained Bi_2_Te_3_ films on PI substrates. The separated triangle dots represent the experimental data and lines are linear fit results using least square method. The Seebeck coefficient *S* is calculated from the fitted slope, of which the standard error is within 0.5%. And the symbol R means that the direction of temperature gradient is reversed.

**Figure 3:**
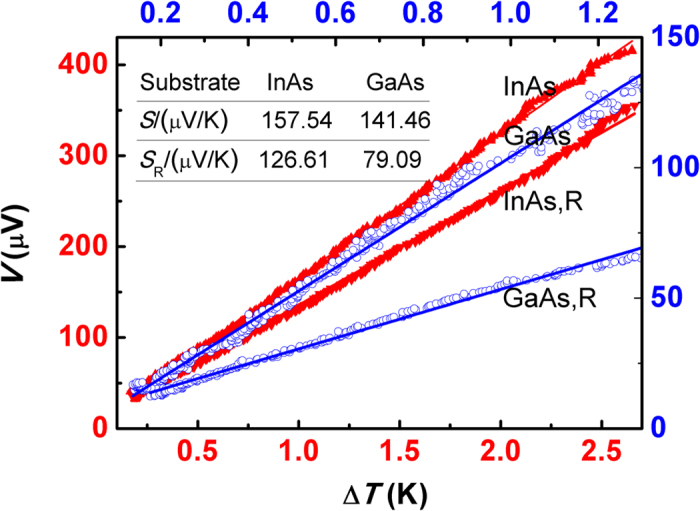
Thermoelectricity of strained Bi_2_Te_3_ films on InAs and GaAs substrates. The separated triangle and circular dots represent the experimental data from the films on InAs and GaAs substrates, respectively. And lines are linear fit results using least square method.

**Figure 4:**
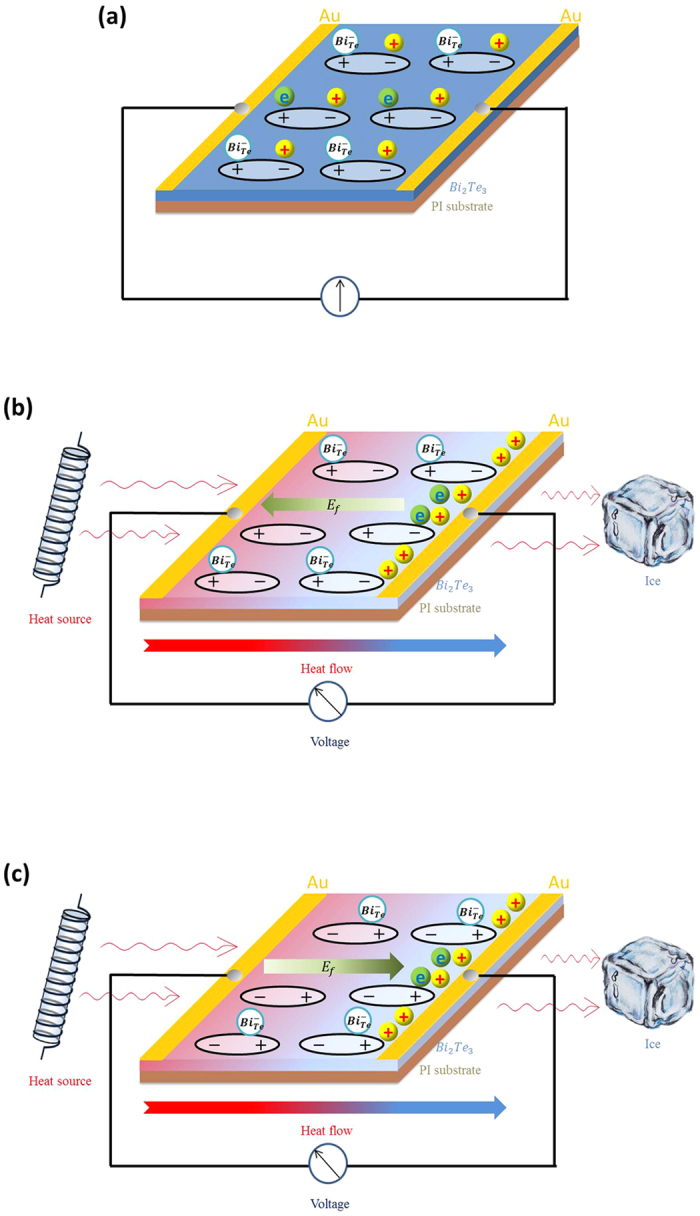
The coupling mechanism of thermoelectric and flexoelectric effects in strained Bi_2_Te_3_ film. (**a**) The diagram illustrates the distribution of carriers and electric dipoles in a strained Bi_2_Te_3_ film without thermal gradient. (**b**) The film has been placed in a thermal gradient field and the thermoelectric field has opposite direction with the flexoelectric field. (**c**) The direction of thermal gradient is reversed compared with (**b**).

**Figure 5:**
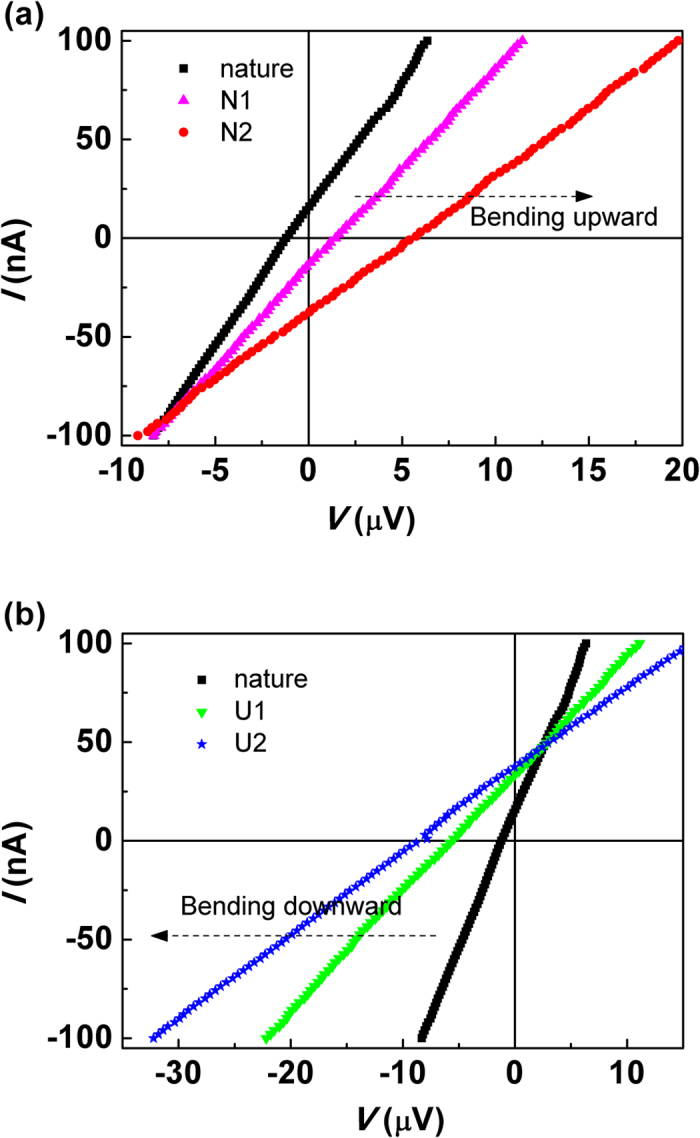
Current-voltage (*I-V*) curves for flexible Bi_2_Te_3_ film under different bending conditions. (**a**) *I-V* curves for flexible Bi_2_Te_3_ film in nature and bending upward state, and N_1_, N_2_ represent the different bending upward degrees. (**b**) *I-V* curves for flexible Bi_2_Te_3_ film in nature and bending downward state, and U_1_, U_2_ represent the different bending downward degrees.
