# Supplementary material for: Anomalous thermoelectricity in strained Bi2Te3 films
Source: Sci Rep. 2016 Sep 7;6:32661. doi: 10.1038/srep32661 (PMC5013394; doi:10.1038/srep32661)
Supplement: Supplementary Information [file srep32661-s1.pdf]

Anomalous thermoelectricity in strained Bi_2Te_3 films

Authors: Yucong Liu[1,5], Jiadong Chen[1,3], Huiyong Deng[1,]*, Gujin Hu[1],

Daming Zhu[4], and Ning Dai[1,2,]*

Supplementary Figures:

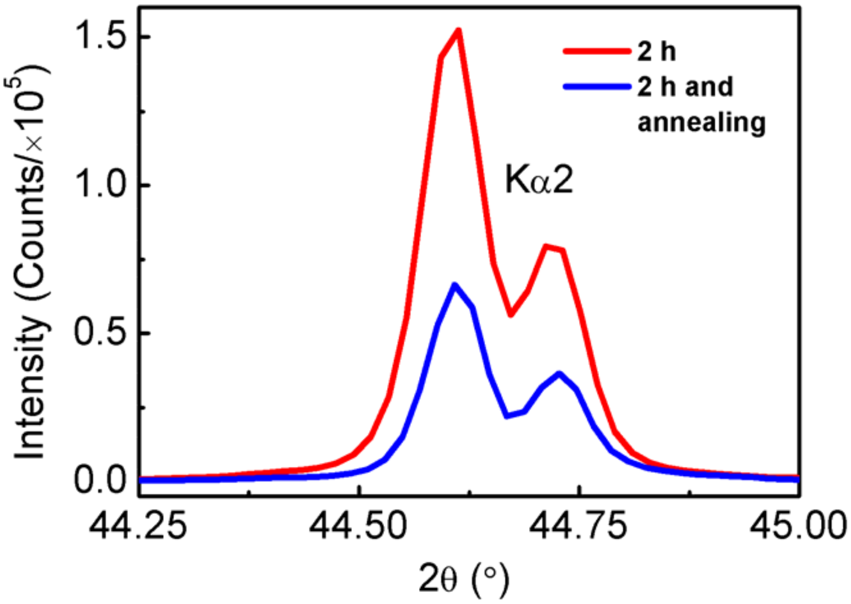

Figure S1 │ XRD patterns of Bi_2Te_3 films on PI substrates. The magnified fine structure of (0,0,15) diffraction peaks.

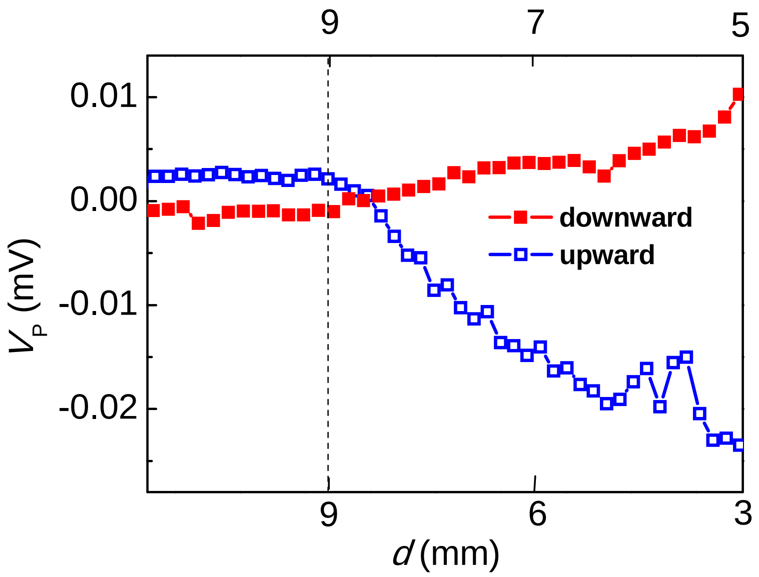

Figure S2 │ Flexoelectricity of Bi_2Te_3 films. The variation of flexoelectric voltage V_f with distance *d* between the two ends of the sample when the film is bent upward or downward.
